# Is short-axis imaging of the LV obligatorily the most accurate method for non-invasive quantitation of the mass of the heart?

**DOI:** 10.1186/1532-429X-11-S1-P255

**Published:** 2009-01-28

**Authors:** Nicholas Farber, Mark Doyle, Ronald B Williams, June A Yamrozik, Rachel Paul, George J Magovern, James A Magovern, Srinivas Murali, Robert WW Biederman

**Affiliations:** Allegheny General Hospital, The Gerald McGinnis Cardiovascular Institute, Pittsburgh, PA USA

**Keywords:** True Mass, Phantom Model, Asymmetrical Geometry, Contiguous Imaging, Thin Slice Imaging

## Introduction

Non-invasive LV mass quantitation is an important clinical metric with considerable ramifications that include substantial morbidity and mortality. Cardiovascular MRI (CMR) has been demonstrated to be the 'gold standard' for the quantification of LV mass for almost two decades chiefly defined by its high spatial resolution affording very low variability. Over the last two decades the technique has evolved typically, into contiguous, 2D short-axis (SA) orientations as the standard for CMR cardiac mass quantitation, believed to be inherently most accurate. However, as compared to other techniques wherein accuracy suffers from inability to define endocardial/epicardial borders, any inaccuracy CMR suffers is now predominantly governed by the basal-plane acquisition whereupon exact discrimination of the AV-valve plane is arduous.

## Hypothesis

We hypothesized that a systematic interrogation of alternative, non-SA imaging geometric acquisitions might now be more accurate for defining, despite lack of truly contiguous imaging, more accurate and reproducible LV mass measurements.

## Methods

An agar-based gel phantom was created representative of the varied cardiac anatomy, complete with tapered apex, non-planar base, trabeculations and often asymmetrical geometry. The weight of the phantom was measured on a high-performance scale. The phantom was scanned under varied orientations: 2D short-axis, 2D long-axis, 2D radial long-axis, and 3D short-axis imaging. For each orientation, slice thicknesses of 5 mm^2^ and 10 mm^2^ were used, while the 3D scan used 1 mm^3^ and 2 mm^3^ thicknesses. All variations of the parameters were scanned using both steady-state free precession (SSFP) and gradient recalled-echo (GRE). Statistical analyses via Students t test were performed to determine variances of the mass of the agar.

## Results

The calculated mass of the agar based on the short-axis orientation was an average of 121% of the true LV mass. The long-axis orientation gave an average of 95% of the true mass. The radial scan gave an average of 96% of the true mass. The 3D scan (1 or 2 mm slice thickness) gave an average of 98% of the true mass. Larger slice thickness caused a significant overestimation of mass in only the 2D long axis orientation. No significant difference existed between the SSFP and GRE scan sequences in this (static) phantom model.

## Conclusion

By default, 3D imaging via a phantom model designed specifically to emulate the natural heterogeneity of the typical LV is inherently the most accurate, especially via thin slice imaging for quantitation of LV mass. However, scan times limit clinical practicality for optimal accuracy. Contrary to the classical thinking that has evolved over the last 2 decades, the radial long-axis orientation shows great promise, as it avoids basal-plane errors associated with the short-axis orientation and has shorter scan times. Incorporation of this strategy inherently overcomes such basal-plane registration errors which exert a *much* greater influence over accuracy than do contiguous slice and interpolation errors. Future validation of this proof of concept study will include *ex-vivo* human heart studies.

